# Retraction: The impact of insecticides and plant extracts on the suppression of insect vector (*Bemisia tabaci*) of Mungbean yellow mosaic virus (MYMV)

**DOI:** 10.1371/journal.pone.0272190

**Published:** 2022-08-03

**Authors:** The PLOS ONE Editors

The *PLOS ONE* Editors retract this article [1] because it was identified as one of a series of submissions for which we have concerns about authorship, competing interests, and peer review. We regret that the issues were not addressed prior to the article’s publication.

AMAS agreed with the retraction. MY, NAR, NAK, AAZ, GAK, MWA, and MA either did not respond directly or could not be reached. HZ, TL, SH, SS, HK, MJA, ATKZ, and YL did not agree with the retraction.
